# Permeance of Condensable Gases in Rubbery Polymer Membranes at High Pressure

**DOI:** 10.3390/membranes14030066

**Published:** 2024-03-06

**Authors:** Karina Schuldt, Jelena Lillepärg, Jan Pohlmann, Torsten Brinkmann, Sergey Shishatskiy

**Affiliations:** 1 Helmholtz-Zentrum Hereon, Institute of Membrane Research, Max-Planck-Str. 1, 21502 Geesthacht, Germany; karina.schuldt@camfil.com (K.S.); jan.pohlmann@hereon.de (J.P.); torsten.brinkmann@hereon.de (T.B.); sergey.shishatskiy@hereon.de (S.S.); 2 Camfil GmbH, Feldstraße 26-32, 23585 Reinfeld, Germany

**Keywords:** thin film composite membrane, condensable gases, gas transport properties, high pressure, CO_2_ permeance

## Abstract

The gas transport properties of thin film composite membranes (TFCMs) with selective layers of PolyActive™, polydimethylsiloxane (PDMS), and polyoctylmethylsiloxane (POMS) were investigated over a range of temperatures (10–34 °C; temperature increments of 2 °C) and pressures (1–65 bar abs; 38 pressure increments). The variation in the feed pressure of condensable gases CO_2_ and C_2_H_6_ enabled the observation of peaks of permeance in dependence on the feed pressure and temperature. For PDMS and POMS, the permeance peak was reproduced at the same feed gas activity as when the feed temperature was changed. PolyActive™ TFCM showed a more complex behaviour, most probably due to a higher CO_2_ affinity towards the poly(ethylene glycol) domains of this block copolymer. A significant decrease in the permeate temperature associated with the Joule–Thomson effect was observed for all TFCMs. The stepwise permeance drop was observed at a feed gas activity of p/po ≥ 1, clearly indicating that a penetrant transfer through the selective layer occurs only according to the conditions on the feed side of the membrane. The permeate side gas temperature has no influence on the state of the selective layer or penetrant diffusing through it. The most likely cause of the observed TFCM behaviour is capillary condensation of the penetrant in the swollen selective layer material, which can be provoked by the clustering of penetrant molecules.

## 1. Introduction

Thin film composite polymeric membranes (TFCMs) have been established as versatile and flexible tools to be used in gas and vapor separation processes [1,2,3]. This type of membrane benefits from a multi-layer structure, which allows the designer to achieve mechanical stability by choosing the appropriate support structure and optimizing membrane performance by applying a thin layer of the selective material that is most suitable for the separation in question. Carbon dioxide separation is among the most important applications, especially in natural and bio-gas purification and in the emerging carbon dioxide separation from combustion flue gas streams and industrial off-gases [4,5,6,7,8,9]. In recent years, a significant amount of membrane material has been developed aimed at the separation of CO_2_/CH_4_, CO_2_/N_2_, and CO_2_/H_2_ gas pairs. Detailed studies on CO_2_ separation by polymer-based membranes at a wide range of temperatures and pressures have been published over the last four decades [10,11,12,13].

Some glassy polymers as materials with selective layers are very promising for CO_2_ separation, but aging phenomena and plasticization affect their permeation properties in a negative way. For TFCMs with glassy separation layers, a loss in permeance of more than 25% was observed [14,15].

While the transport of gases through rubbery polymeric membranes at low pressures is well studied, only a few publications have addressed the subject of transport behavior at high pressures [16,17,18,19,20,21]. At low pressures with limited swelling, the transport behavior is well described by the Free Volume model (FVM) [22].

It was found that at an average pressure p(feed)-p(permeate) not exceeding 20 bar, the permeation of a complex gas mixture is adequately described by the FVM using model parameters derived from results of simple, single gas experiments [22,23,24,25,26]. However, above a certain pressure difference, the transport of CO_2_ did not follow the expected tendency. This has initiated experimental work concerning the investigation of single and mixed gas permeation using, as it is considered to be well studied, PolyActive^TM^ 1500 TFCMs [2,27,28,29]. The results showed that a clear pressure threshold exists below which the FVM can describe the transport of multicomponent gas mixtures with an accuracy that is sufficient for practical separation process designs. Above the threshold, the CO_2_ permeance was significantly higher than the values predicted by the FVM.

An investigation of condensable gas transport through thick polymer films or integral asymmetric membranes in the full range of pressures has only been conducted a few times, for example, in the work of Favre et al. [30]. This study shows no unexpected changes in the permeation curve for highly soluble penetrants such as chloroform, 2-butanol, 1-butanol, etc., in the full range of gas activity, defined as the ratio of partial and saturated vapor pressures at a given temperature. In the case of microporous materials, where condensation of penetrants is possible and capillary or selective surface flow can occur, the shape of the permeation curve is very complex [31,32].

This paper describes the experimental results of permeation behavior investigations of two single condensable gases, CO_2_ and C_2_H_6_, in three different rubbery polymers serving as the selective layer for TFCMs in the full range of pressures up to the point of gas condensation on the feed side of the membrane. For the systematic investigation of the permeation behavior of gas separation membranes, a special laboratory-scale test facility for high pressures was developed.

The main emphasis was put on the investigation of CO_2_ behavior in PolyActive^TM^ 1500 TFCM due to the envisaged membrane applications for CO_2_ separation from various gas streams [2,24,33,34]. In order to find out whether the observed facts are specific to only the CO_2_/PolyActive^TM^ pair or if a similar behavior of condensable gas and rubbery polymer can be observed for other combinations, ethane as an alternative condensable gas and two siloxane-based polymers, polydimethylsiloxane and polyoctylmethylsiloxane, were used in the experiments. Helium was used to prove the absence of porosity in the prepared TFCM samples and to prove that the designed experimental facility delivers adequate results. The experiments were performed in a pressure range of 1–65 bar at temperatures of 10–34 °C.

## 2. Materials and Methods

The TFCM selective layers were made from the rubbery polymers PolyActive™ 1500 (PolyVation BV, Groningen, The Netherlands), composed of 77 wt% poly(ethylene glycol) soft segments and 23 wt% poly(butylene terephthalate) hard segments [35] (PA), polyoctylmethylsiloxane (POMS) (abcr GmbH, Karlsruhe, Germany), and polydimethylsiloxane (PDMS) (the supplier cannot be disclosed due to licensing limitations).

The gases had a purity of at least 99.95% for C_2_H_6_, 99.996% for He (both Air Liquide Deutschland GmbH, Stelle, Germany), and 99.995% for CO_2_ (Linde GmbH, Leuna, Germany).

### 2.1. Preparation of Samples for Gas Sorption Experiments

The samples for gas sorption experiments were prepared as follows: The 3 wt% solution of PA was prepared in tetrahydrofuran (for analysis grade, Merck KGaA, Darmstadt, Germany) and stirred under reflux for at least 2 h until full polymer dissolution. To prepare a thick isotropic film, an Al cylinder with a polished bottom surface was placed on a leveled Teflon™-coated glass plate. The polymer solution was poured into the formed vessel; and the solvent was evaporated under a slow N_2_ flow for 48 h at 25 °C, as described in [36]. The 6 wt% PDMS solution in iso-octane was stirred for 2 h under ambient conditions and poured into a Teflon™ beaker with a leveled bottom. The solvent was left to evaporate for 72 h at ambient conditions under a constant N_2_ flow. All samples were treated in a vacuum oven at 70 °C for at least 3 h in order to remove the residual solvent. To complete the crosslinking reaction in the PDMS sample, it was additionally exposed to 100 °C for 2 h in a vacuum oven.

### 2.2. TFCM Samples for Gas Transport Experiments

The TFCMs used in the current study contained a selective layer consisting of PA, PDMS, or POMS. The PA selective layer was deposited on a gutter layer of crosslinked PDMS, which in turn was formed on a porous ultrafiltration (UF) polyacrylonitrile (PAN) membrane supported by a polyester nonwoven, which provided the mechanical strength. A protective layer of PDMS was deposited over the PA selective layer to cover possible defects (pin holes) and ensure membrane protection [37]. This membrane is known for its high CO_2_ permeance, with the selectivity of the TFCM close to that of the selective layer material [2]. To compare the gas transport properties determined at various pressures and temperatures, TFCMs with PDMS and POMS selective layers on the PAN UF membrane were studied as well. The list of membranes is presented in Table 1.

### 2.3. Gas Permeance Measurements up to and beyond the Saturated Vapor Pressure of a Penetrant

The single gas permeances of TFCM samples were determined using a “constant pressure/variable volume” (PI) facility developed at Helmholtz-Zentrum Hereon and described elsewhere [24,39,41]. PI measurements conducted at a constant temperature and low pressure were also used for the estimation of Knudsen-type dependence for the porous UF-PAN membranes.

For high-pressure experiments, a special experimental setup was developed that allows automatic measurements in wide pressure and temperature ranges. The setup utilizes the “single gas experiments at high pressures” method of gas transport experiments and is described elsewhere [24]. This setup was designed for experiments with high-performance flat sheet gas separation membranes. The immersion of the membrane test cell into the water tank of a cooling thermostat Huber MPC-K20 (HUBERLAB AG, Aesch, Switzerland) made it possible to carry out experiments at temperatures in the range of 5 to 60 °C. The whole setup can be used for experiments at feed pressures ranging from 1.5 to 100 bar abs with the pressure on the permeate side of the membrane close to ambient.

The conduction of automated measurements is realized using an electrically adjustable pressure reducer (B.E.S.T. Fluid Systems GmbH/Swagelok Hamburg, Brackel, Germany) that controls the feed pressure. After the pressure reducer, the gas line becomes a 20 mm in diameter test cell, which is placed in the water tank of a thermostat. The thermostat ensures that the test cell has a defined and constant temperature. Before and after the test cell, the pressure sensors LEO 3 (Keller Druckmesstechnik GmbH, Jestetten, Germany) are installed to control the feed and permeate pressures during the dead-end experiment. For the recording of the permeate flow, the experimental setup was equipped with four mass flow sensors SLA5800 Series (Brooks Instrument GmbH, Dresden, Germany) with gas flow ranges of 0–5, 0–50, 0–500, and 0–5000 cm^3^ (STP) min^−1^. Since the sensors are calibrated with N_2_, the results for each gas were adjusted using a gas factor provided by Brooks Instrument GmbH. The flow sensors were combined “in series” with the “smallest” flow sensor first, and such an arrangement enabled the experiments to be conducted in the full range of pressures for every gas under study. To take into account the effect of the flow resistance of the smallest flow sensor, a pressure sensor with a pressure range of 0–4 bars was installed immediately at the permeate exit of the measurement cell, and 6 mm stainless steel tubes were used for connections on the permeate side. The chosen design allows one to work with permeate flows up to 2500 cm^3^ (STP)/min, which is in the range of a laminar-type flow in the permeate side tubing. The schematic design of the test cell is given in Figure S1.

In order to control changes in membrane performance that could be caused by penetrants, highly soluble in the material of the selective layer, each measurement sequence was started and ended with the inert gas He. Before the measurement was taken, the experimental setup was flushed with the gas to be measured. The measuring program was applied in such a way that the system was first brought to and stabilized at the desired temperature for at least 10 min. Afterwards, the specified pressure curve was run through. For each measuring point, the pressure and temperature on the feed and permeate sides, as well as the permeate flow rate, were recorded automatically.

The temperature sensor Pt100 with a 500 µm diameter was installed into the porous sintered metal disc in direct contact with the permeate side of the membrane. It enabled monitoring the temperature of the gas permeating through the membrane at a location that was as close as possible to the permeate side of the selective layer. Long, 300 s pressure equilibration times and high permeate flow rates through the membrane were sufficient for temperature equilibration on the permeate side of the membrane. Since Pt100 was installed in the center of the sintered metal support, it was assumed that the measured temperature would be dominated by the temperature of the gas flowing through the membrane and that there would be a negligible influence of the heat coming from the thermostat bath through the bulky stainless steel body of the measurement cell and finally through the highly porous sintered metal disc.

Gas transport experiments were carried out for membrane samples with an effective membrane area of 1.72 cm^2^. Samples of 20 mm in diameter were placed into the measurement cell on a sintered metal support with an embedded temperature sensor. The membrane was sealed with an EPDM O-ring. The measurement cell was temperature-equilibrated for 1200 s after the thermostat reached the desired temperature. The initial temperature of the experiment was always chosen to be 10 °C. After the temperature equilibration step, feed pressure was applied to the sample, starting from the lowest pressure point possible for the pressure regulator. The pressure was equilibrated for 300 s at the achieved pressure, and after this, the flow rate data were acquired from the mass flow sensors, and the next pressure value was set. In total, 38 pressure points were acquired, covering the full possible range of gas pressures. The highest possible pressure for both CO_2_ and C_2_H_6_ was in accordance with the gas bottle temperature, which was kept in a temperature-stabilized lab. Such an arrangement allowed, in some cases, to overcome the condensation pressure of the gas under study at the measurement cell temperature of 10 °C, as will be shown later. After reaching the highest pressure setting, the experiment was continued with a stepwise decrease in the feed pressure down to the initial feed pressure setting. This allowed us to investigate possible hysteresis in the gas permeance for feed pressure, increasing and decreasing parts of the experiment. After the minimum pressure was reached, the temperature of the measurement cell increased by 2 °C and the experiment was repeated. In the current publication, experimental data mainly at 10, 20, and 30 °C are demonstrated, as well as a series of measurements at temperatures between 10 and 34 °C in 2 °C steps.

The permeate pressure was changing insignificantly during the experiment. The highest observed permeate pressure was 1.2 bar abs for the case of the highest observed permeance of 14 m^3^(STP) m^−2^ h^−1^ bar^−1^. The change in the permeate pressure is related to the resistance to the gas flow caused by the “smallest” mass flow sensor installed on the permeate side of the experimental facility. The increase in the permeate pressure by 0.2 bar when the applied feed pressure was above 50 bar was considered insignificant to cause changes in the gas flow through the membrane.

### 2.4. Gas Sorption over a Wide Pressure Range

Gas sorption measurements of pure gases were performed in an experimental system equipped with a magnetic suspension balance (MSB) (TA Instruments, Eschborn, Germany). The system includes a thermostat (Julabo GmbH, Seelbach, Germany), a gas supply, and a vacuum pump (Pfeiffer Vacuum GmbH, Aßlar, Germany). The MSB allows the continuous determination of the mass of sorbent materials from 10 mg to 10 g. Auxiliary equipment such as a titanium sinker permits an estimation of the density of the fluid in situ. The adsorption isotherms of pure CO_2_ in thick films were acquired in a temperature range from 10 °C to 30 °C, with recordings of the sample masses, temperatures, and pressures every 5 s. The density of the fluid was estimated every 5 min. Samples were degassed at ambient temperature under a vacuum for 24 h to remove residual solvents and pre-adsorbed gases.

Densities of thick isotropic films required for the evaluation of gas sorption experiment results were estimated by Archimedes’ principle using the analytical balance Excellence Plus XP105DR (Mettler-Toledo GmbH, Gießen, Germany), density determination kit, and auxiliary liquid FC-770 (3M, Saint Paul, MN, USA). For each material, five pieces of a sample were weighed for an accurate determination of the density and experimental error. A description of the density estimation is given in Appendix A.

### 2.5. Experimental Uncertainties

The uncertainties of the equipment used in the current study are presented in Table 2.

## 3. Results and Discussion

### 3.1. Experimental Results

#### 3.1.1. Quality Validation of the Experimental Setup and TFCM Stability in the Available Pressure Range

**Figure 1:**
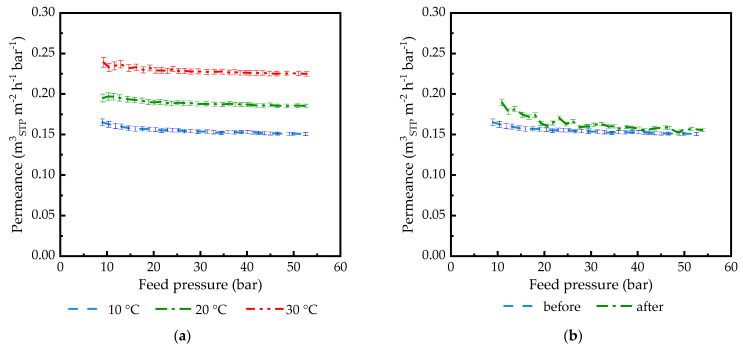
He permeance of PDMS1280-PAN TFCM plotted against feed pressure: (**a**) permeance in the pressure range 5–50 bar at 10, 20, and 30 °C; (**b**) comparison of membrane permeance at 10 °C before and after measurement session using condensable gases. Experimental uncertainty was calculated using linear error propagation.

Before the experiments for the determination of membrane permeance subjected to the high activities of condensable gases, it was necessary to test the functioning of the experimental facility in a wide pressure range. To investigate the effect of pressure on gas transport through TFCMs with a rubbery polymer-based selective layer without taking into account the interaction between the penetrant and membrane material, experiments with He as a permanent, noble gas that showed allowable solubility were conducted. Figure 1a shows the results of helium transport measurements for PDMS1280-PAN TFCM in the pressure range up to 55 bar for 10 °C, 20 °C, and 30 °C. For each temperature, no significant change in the permeance with increasing pressure was observed. The same was observed for membranes with other selective layer materials. Hence, there is no link between the mechanical pressure applied to the membrane and the transport behavior of the tested rubbery membranes. No significant compaction could be detected if the experiments were carried out with a permanent gas under the chosen measurement conditions. In Figure 1b, two curves are compared, which show He permeance before and after the full set of experiments carried out for this membrane sample with condensable gases. The similarity of the two measurement curves proves that the membrane did not significantly change its properties when exposed to high activities of condensable penetrants. The experimental facility was proven to deliver reliable experimental results at the chosen pressures and temperatures.

#### 3.1.2. Sorption of CO_2_ in PDMS and PA Isotropic Films

**Figure 2:**
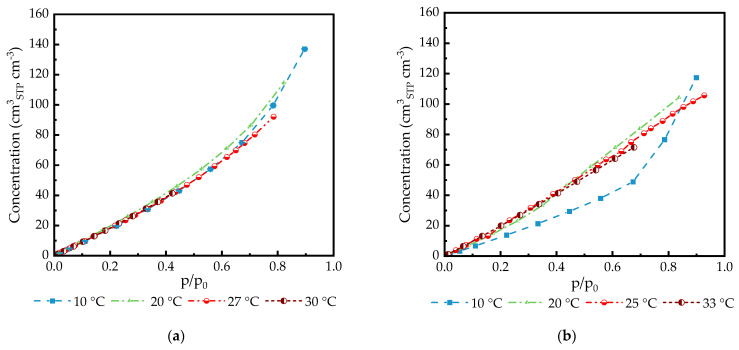
Sorption of CO2 in isotropic films of (**a**) PDMS films 261 µm (at 10 °C, 20 °C, and 27 °C); 283 µm (at 30 °C); (**b**) PA films 388 µm (at 10 °C, 20 °C, and 33 °C), and 173 µm (at 25 °C). The experimental uncertainty is less than 0.05%, and the standard deviation in each measurement point represents a set of more than 100 data points. Some error bars are smaller than the symbols.

Sorption of CO_2_ in PDMS and PA was carried out at temperatures from 10 °C to 30 °C and at pressures up to p/po = 0.9 (Figure 2). Higher pressure was not applied to the samples due to the danger of gas condensation within the measurement equipment. 

Experiments with C_2_H_6_ were not carried out due to technical issues. Nevertheless, both PDMS and PA were numerously investigated for gas sorption in a wide range of pressures, and all published isotherms were adequately described by mathematical models applicable to these materials [42,43,44,45].

The density levels of PDMS and PA, necessary for evaluating the data acquired during the gas sorption experiment, were determined using two methods utilizing the Archimedes principle. In one case, the density was determined using perfluorinated FC770 as the fluid with a large molecule size, thus reducing the probability of molecule diffusion in the polymer sample (Appendix A); in the second case, the density was determined in He within the magnetically suspended microbalance. He is an inert gas with a density that allows for good resolution of the sample weight difference between values measured in a vacuum and in the He atmosphere. The density values of isotropic films of PDMS and PA are listed in Table 3.

In the pressure range up to p/p_o_ = 0.6, isotherms can be adequately described using Henry’s law for both PDMS and PA. The influence of PDMS swelling in this pressure range is not significant. A swelling degree of more than 10% will be expected at pressures higher than 50 bar for a non-crosslinked polymer [46,47,48].

For the PA thick film, a linear increase in CO_2_ uptake was observed for temperatures from 25 °C to 33 °C (Figure 2b). The non-linearity behavior was observed for the temperature of 10 °C, which is below the crystallization temperature of poly(ethylene glycol) domains of PA reported as 25–28 °C [49,50].

Numerous publications on gas sorption in rubbery polymers have always demonstrated a continuous dependence of the quantity of gas dissolved in the polymer on the applied pressure, which can be described by the Henry-type isotherm in cases of insignificant interaction of the solute with the polymer matrix or by Flory–Huggins-type isotherms in cases where the solute/polymer interaction is significant and an increased concentration of the solute in the polymer causes swelling of the polymer [30,51]. In this case, swelling can lead to changes in the diffusion coefficient, as was clearly shown by, e.g., Lin and Freeman. They studied the sorption of various condensable gases in semi-crystalline poly(ethylene oxide) [44,52].

#### 3.1.3. Gas Flow Rate through TFCMs and Isotropic Films of PDMS and PA

**Figure 3:**
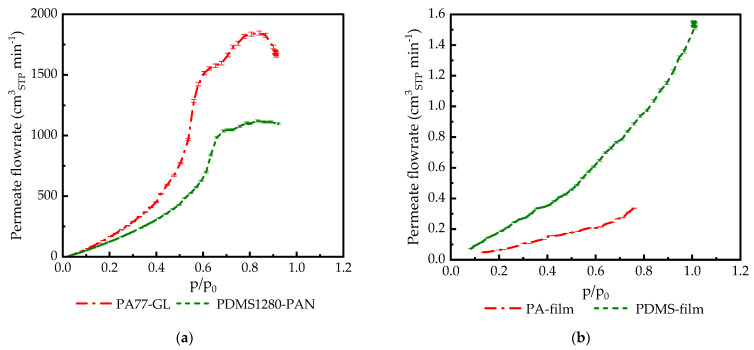
CO_2_ permeate flow rate at 20 °C for (**a**) PA77-GL and PDMS1280-PAN; (**b**) PA 19 µm and PDMS 285 µm isotropic films.

The measurements of condensable gas permeances of TFCMs in the full activity range showed that the shape of the flow rate/feed pressure (activity) curve was very far from expectations based on observations reported in the literature. As shown in Figure 3a, the shape of the permeate flow curve is not monotonous, in contrast to the same parameter determined for thick films (Figure 3b). 

Experiments with thick isotropic films of PA and PDMS on the experimental setup used in the current study presented certain difficulties because the setup was designed for experiments with mechanically robust TFCMs with extremely small selective layer thicknesses. Isotropic films of PA and PDMS with thicknesses of 19 µm and 285 µm, respectively, were not sufficiently mechanically stable to be compressed with the standard O-ring used for membrane sealing in high-pressure experiments. The ethylene-propylene-diene(monomer) rubber (EPDM) O-ring with the Shore 70 hardness was used for experiments with polymer films, and the measurement cell was closed with the lowest possible pressure applied to the membrane to reduce O-ring intrusion into the polymer film and thus prevent changes in the film shape within the cell due to compression. The experiments were carried out at 20 °C only and with CO_2_ only. C_2_H_6_ caused film damage already at low feed pressures. The PDMS film withstands CO_2_ in the full range of CO_2_ activity when the feed pressure is gradually increased and breaks when the feed pressure begins to be reduced. PA film withstands CO_2_ only up to an activity level of 0.75. Both films showed the presence of significant swelling when removed from the measurement cell, and vertically oriented wrinkles broken on the top were observed for both polymers.

It is interesting to note that for PA77-GL and PDMS films, multiple experimental points were collected at the highest pressure. The positioning of these points on top of one another indicates that the sample under investigation is in equilibrium and that it does not change permeance in time, only under changing pressures.

#### 3.1.4. Investigation of TFCM Permeance in Relation to the Feed Pressure of Condensable Penetrants

**Figure 4:**
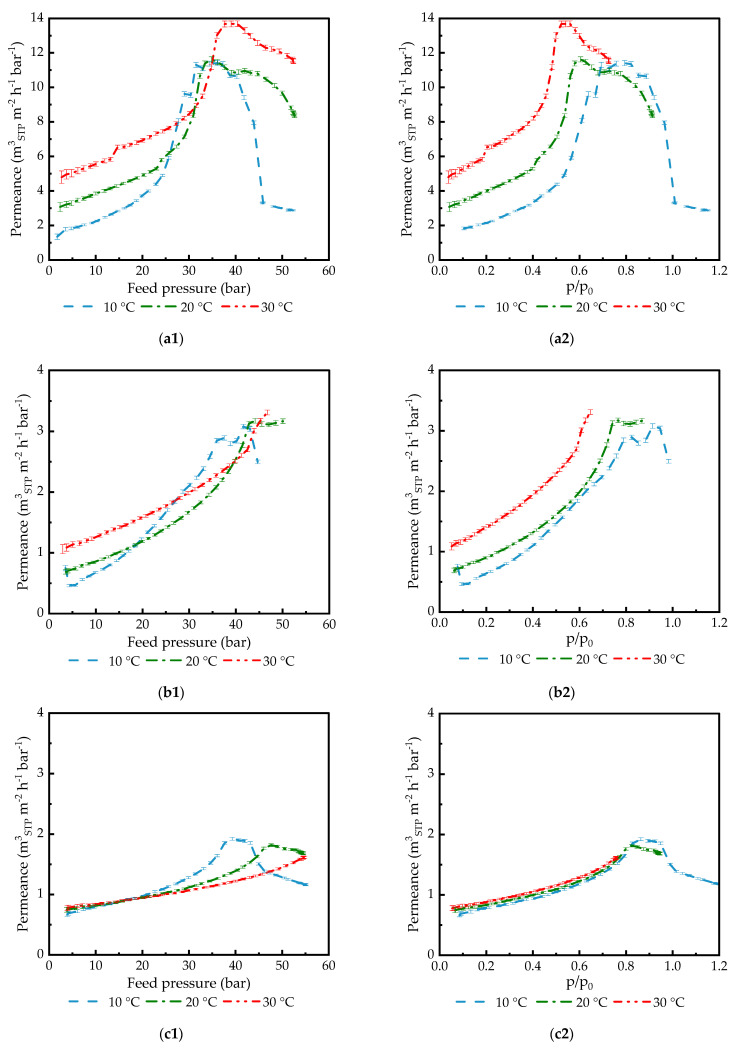

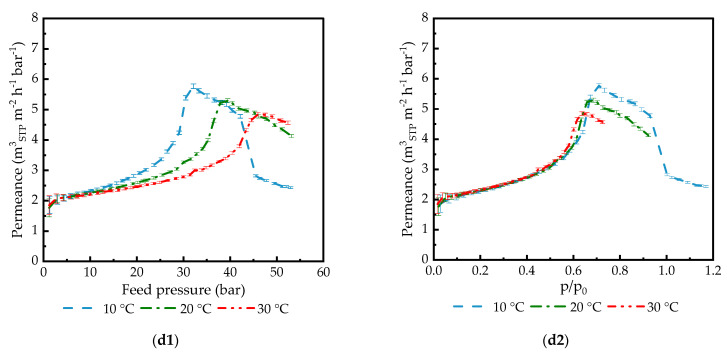
CO_2_ permeance of various TFCMs measured at 10 °C, 20 °C, and 30 °C: (**a1**) PA77-GL vs. feed pressure; (**a2**) vs. activity; (**b1**) PA186-GL vs. feed pressure; (**b2**) vs. activity; (**c1**) POMS6250-GL vs. feed pressure; (**c2**) vs. activity; (**d1**) PDMS1280-PAN vs. feed pressure; (**d2**) vs. activity. The feed pressure increasing part of the experiment is shown for better figure clarity.

**Figure 5:**
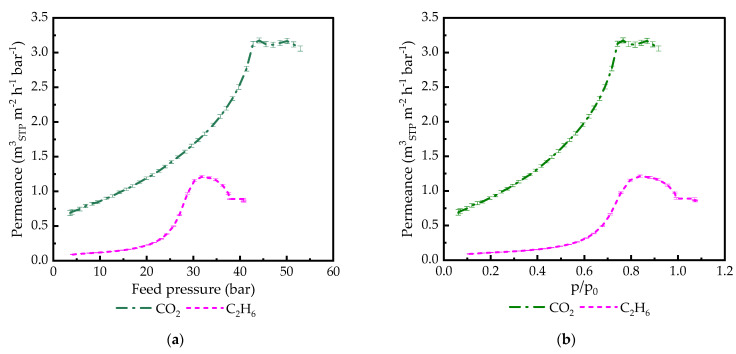
CO_2_ and C_2_H_6_ permeance in PA186-GL TFCM plotted: (**a**) vs. feed pressure measured at 20 °C; (**b**) vs. penetrant activity at 20 °C. Feed pressure increasing part of the experiment is shown for better figure clarity.

The experiments with various TFCMs listed in Table 1 were carried out in the full possible range of pressures. At the experimental temperature of 10 °C, it was possible to overcome the saturation pressure since the gas bottle was kept in the lab with the temperature stabilized at 20 °C. This allowed for the investigation of membrane behavior under the influence of a liquid penetrant applied to the feed surface of the TFCM. Since the experiment contained pressure increasing and decreasing parts, it was possible to observe whether irreversible changes to the membrane could be introduced by the application of a high-pressure gaseous or even liquid penetrant. No significant deviation between the two curves corresponding to the pressure increasing and decreasing parts of the experiment was found, indicating that the chosen experimental conditions allowed for membrane property stabilization after the pressure was changed. Only the pressure increasing parts of the experiments are depicted in Figure 4 and Figure 5.

#### 3.1.5. Gas Transport Properties of GL and UF-PAN Membranes Used as Supports for TFCMs

**Figure 6:**
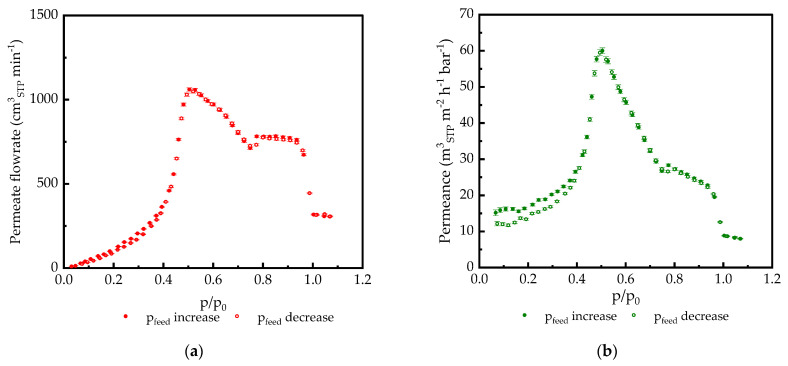
CO_2_ Transport parameters at 20 °C plotted against feed CO_2_ activity for GL used as a standard gutter layer support: (**a**) permeate flowrate; (**b**) permeance. Data points related to the feed pressure *p_feed_* increasing and decreasing parts of the experiment are demonstrated.

**Figure 7:**
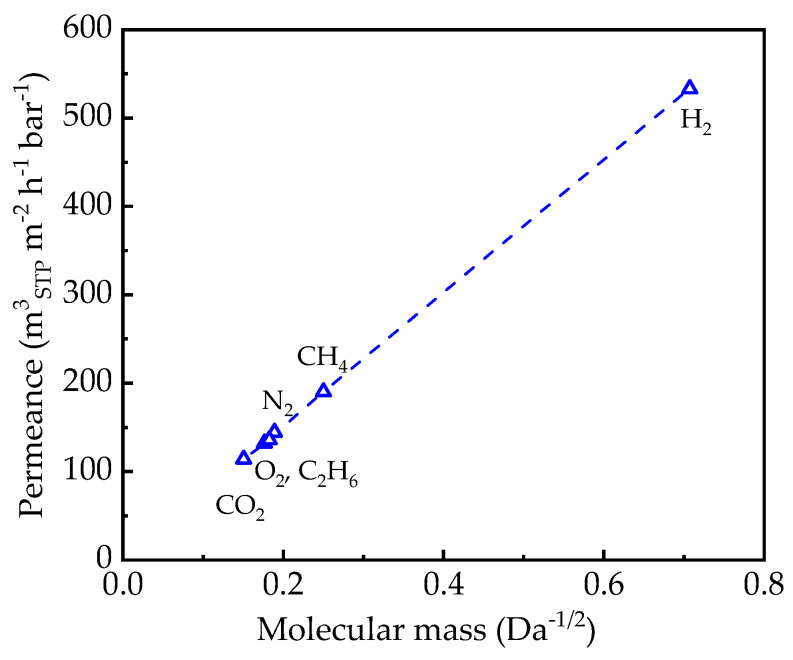
Knudsen-type dependence of gas permeance determined for the UF-PAN membrane at 25 °C for H_2_, CH_4_, N_2_, O_2_, CO_2_, and C_2_H_6_ at a feed pressure of 170 mbar abs.

Membranes used as supports for the formation of selective TFCMs were tested for gas transport properties in order to investigate a possible influence of the support on the resulting gas transport properties of TFCMs. Both GL (Figure 6) and UF-PAN (Figure 7) membranes demonstrate permeances that are significantly higher than that determined for selective TFCMs. 

According to the resistance model introduced by Henis and Tripodi [53] for the analysis of gas separation membrane performance, the selective layer of TFCM governs the gas transport properties of TFCM.

The measurement of UF-PAN was carried out only at as small a feed pressure as possible due to the very high membrane permeance. The gas transport properties of the UF-PAN membrane can be described by the Knudsen-type gas flow through porous media (Figure 1) [54].

The GL membrane demonstrates the presence of the peak in the CO_2_ permeance curve already at ca. 0.5 feed CO_2_ activity with a significant permeance decrease in the activity range 0.5–1.0.

#### 3.1.6. Changes in Permeate Temperature during Experiments in the Full Range of CO_2_ Activity

**Figure 8:**
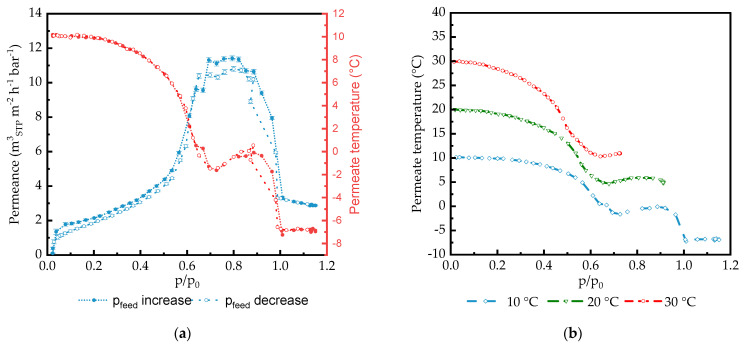
(**a**) Permeance (blue line) and permeate temperature (red line) vs. CO_2_ feed activity for PA77-GL TFCM acquired for feed temperature 10 °C, and filled and open symbols show feed pressure increasing and decreasing parts of the experiment, respectively. (**b**) Permeate temperature at feed pressure increased in part of the experiment carried out at feed temperatures of 10 °C, 20 °C, and 30 °C.

**Figure 9:**
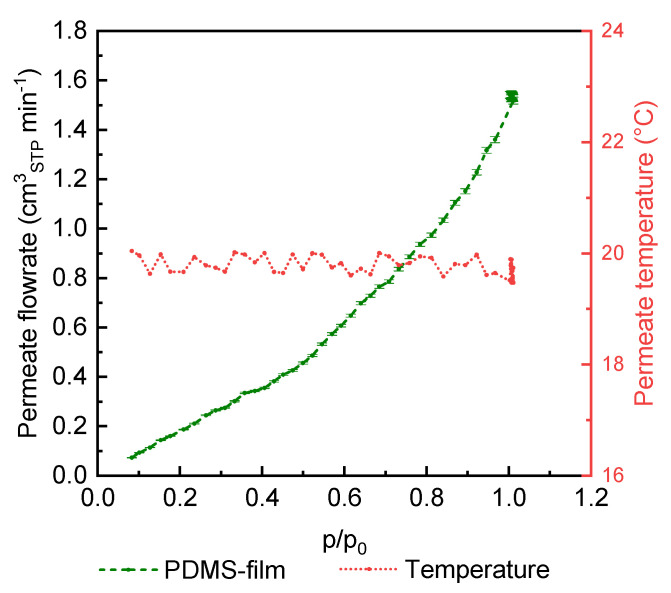
CO_2_ permeate flowrate and permeate temperature obtained at 20 °C feed temperature for PDMS film.

Permeate temperature was controlled using temperature sensor placed immediately at the permeate surface of the membrane under study. Figure 8 and Figure 9 demonstrate clear relation between peak in permeance and change of the permeating gas temperature.

### 3.2. Analysis of the Experimental Results

#### 3.2.1. Experiments with Thick Isotropic Films

The experimental results obtained for thick isotropic PDMS and PA films are presented in Figure 2, Figure 3 and Figure 9.

CO_2_ sorption data obtained in the CO_2_ activity range 0–0.95 show no unusual behavior of the polymer/gas pair. Isotherms for different temperatures are very similar for one polymer. While CO_2_ isotherms for PDMS follow the Flory–Huggins behavior typical for rubbery polymers [45], PA isotherms can be described as simple Henry isotherms at temperatures over the melting point of poly(ethylene glycol) at 25 °C. The beginning of the crystallization process can be seen at 20 °C. At 10 °C, poly(ethylene glycol) blocks of the PA block copolymer are in a highly crystalline state, and CO_2_ starts to act as a plasticizer, reducing the melting temperature and promoting crystallites melting at activity levels of 0.7 and above [50]. At the highest activity, CO_2_ solubility in PA is the highest, as presented in Figure 2b, and this is in clear accordance with the theory of gas transport, though polymers were introduced first by Barrer and Rideal in 1939 [55,56]. Only small differences in the isotherms acquired at different temperatures are in agreement with the low values of CO_2_/polymer partial enthalpies of sorption that have been numerously reported elsewhere [57,58,59].

Gas transport experiments carried out in the full possible range of condensable gas pressures did not show deviations from the permeance/pressure relationship expected for rubbery polymers. The slightly non-linear shape of the CO_2_ flow curve can be explained by the limited swelling of the polymer by the penetrant, leading to an increase in permeance at higher pressures. Unfortunately, the PDMS film did not withstand the decrease in the permeate pressure, and the permeance data were not collected for the pressure decreasing part of the experiment. The PA film, as stated above, did not withstand an increase in the CO_2_ pressure above an activity of p/p_o_ = 0.75. After the experiment, the measurement cell was opened and the states of both the PDMS and the PA films were investigated. It was found that vertical wrinkles were formed with cracks on the highest point of the wrinkle. Such a change in the film’s geometry can only be attributed to the swelling of the polymer by the penetrant, as no other influence capable of causing a change in the film’s geometry can be identified.

#### 3.2.2. TFCM Permeance Peak at High Pressures

As mentioned above, during the work on membrane gas separation of complex gas mixtures using PA TFCM [24], it was found that the FVM cannot adequately predict CO_2_ transport at elevated partial pressures. The model gave a CO_2_ permeance lower than the experimental value, and the deviation increased with a partial pressure increase.

To investigate this phenomenon in the full range of partial pressures up to and over the saturation pressure of a penetrant, the transport of condensable gases through rubbery polymer-based TFCMs was studied for membranes with selective layers made of different materials, as presented in Table 1. The PA TFCM was developed for the separation of CO_2_ containing gas mixtures, with the main emphasis on the separation of CO_2_ from flue gas of various origins. This consideration led us to the decision to conduct extensive experimental work with CO_2_, while C_2_H_6_ was used for comparison purposes. The results of the experiments with different membranes for pressures up to 65 bar are shown in Figure 3, Figure 4, Figure 5, Figure 6 and Figure 9. All the tested rubbery TFCMs show a strong increase in permeance with increasing feed pressure. Up to about 20 bar, the observed increase is as expected for membrane material swollen by the penetrant, but above this pressure, the curve shape becomes more complex, as with an exponential increase. The curve shows a peak followed by a small plateau, followed by a steady permeance decrease up to the point of gas condensation. The peak position, plateau width, and overall curve shape depend on the selective layer material and membrane morphology. 

Basically, the transport of small molecules through rubbery membranes is driven by the fugacity difference in accordance with the solution/diffusion mechanism [60]. Hence, the gas flow rate through the membrane is determined by several factors: the adsorption of gas on the feed membrane surface; the diffusion of dissolved gas molecules through the bulk of the selective layer; and finally, the desorption on the permeate side of the selective layer. The transfer of the penetrant from the selective layer into supporting layers depends on the resistance of these supporting layers to the penetrant flow; it can either be the porous surface of PAN with surface porosity not more than 13% or a gutter layer formed of highly permeable and low selective PDMS, which provides a smooth surface for the selective layer deposition and effective drainage of the penetrant from the whole permeate surface of the selective layer into the porous structure of the UF-PAN membrane.

The quantity of diffusing gas molecules Is determined by the solubility coefficient of the gas under study in the selective layer material. This parameter can be directly acquired from the results of the gas sorption experiment. The corresponding isotherms are presented in Figure 2. 

While the respective membrane material directly influences the dissolution and desorption of gas molecules during the gas transport process, the diffusion within the selective layer can be affected by the degree of swelling caused by the penetrant interaction with the selective material. The sharp increase in permeance beyond that described by the FVM indicates that as the concentration of the dissolved molecules in the selective layer increases, either the solubility or the diffusivity of the penetrant in the polymer increases significantly. One can speculate that in addition to polymer swelling as a possible cause of the permeance increase, the effect of the penetrant–penetrant molecules’ interaction confined in the free volume voids of the polymer matrix starts to be significant, even overcoming the effects of the penetrant–polymer interactions [61,62]. The penetrant–penetrant interaction can also lead to a negative, flow-reducing effect since penetrant molecule clustering becomes more probable, resulting in an increase in the effective diameter of the penetrant and a consequent mobility decrease in the sorbed molecules [63]. The cluster formation leads to a decrease in the diffusion coefficient, and it has already been detected for carbon dioxide [64]. The effects of swelling and clustering have opposite effects regarding the permeate flow rate. Due to the different interactions of CO_2_ with the selective membrane materials under study, the shape of the curves presented in Figure 4 varies for each material.

Figure 4 presents the data collected for CO_2_ permeance in TFCMs with selective layers made of different polymers (PA, PDMS, and POMS) and different selective layer thicknesses. For each gas/TFCM pair, the following two plots are presented: one for permeance plotted against feed pressure, and another for penetrant activity. As one can see, plots with penetrant activity give more generalized information. For two polymers with no specific interaction between the polymer and CO_2_, the peak value of permeance for all three temperatures is reached at the same penetrant activity, ca. 0.83 for POMS6250-GL and ca. 0.66 for PDMS1280-GL. PA77-GL and PA186-GL give a different picture: the position of the permeance peak shifts to a lower CO_2_ activity value with increasing temperature. It is important to mention that the maximum permeance value at 10 °C in the case of PA77-GL is very similar to that at 20 °C, indicating the melting of poly(ethylene glycol) crystallites under the influence of high CO_2_ pressure.

The difference in the peak position between two PA-based membranes indicates that the thickness of the selective layer is important for the observed effect. At smaller selective layer thicknesses, peak permeance is reached at lower penetrant activity.

After the peak, the permeance of PA77-GL started to decrease in terms of the gas flowrate through the membrane (as in Figure 3a), which became mostly independent of pressure. One can presume that at this state of the polymer/penetrant system, the maximum possible amount of penetrant is dissolved in the polymer matrix, further swelling is not possible anymore, and increases in the pressure (activity) are not followed by a proportional increase in the penetrant flowrate. Another possibility is changes in the penetrant state in the polymer matrix, e.g., clustering, as discussed above. 

The curve corresponding to the PA77-GL permeance at 10 °C shows a decreasing trend of CO_2_ activity above 0.8 (Figure 4(a2)) and follows it to the point of feed gas condensation. At a CO_2_ feed activity of 1, the trend changes to a mostly horizontal line. At this moment, the experiment on gas transport turns to the experiment of liquid CO_2_ pervaporation through the polymeric membrane. The flowrate of the penetrant is not dependent anymore on the pressure. As will be discussed later, the presence of liquid CO_2_ on the feed membrane surface does not influence membrane integrity, and experiments involving gas condensation were repeated numerous times and clearly demonstrated that the investigated polymeric membranes are not altered by the liquid penetrant.

In order to find out whether the observed effect is only related to CO_2_ or if it is common for condensable gases, experiments with C_2_H_6_ were carried out with PA186-GL. As follows from Figure 5, the system PA/C_2_H_6_ demonstrates the same behavior as PA/CO_2_ in the full range of C_2_H_6_ activity. The C_2_H_6_ permeance reached its peak value at 0.85 activity of C_2_H_6_. The peak permeance of C_2_H_6_ was significantly lower than that of CO_2_. After C_2_H_6_ condensation on the feed membrane surface, the permeance did not show dependence on pressure. As in the case of CO_2_, the membrane successfully survived exposure to the liquid penetrant on its surface.

**Figure 10:**
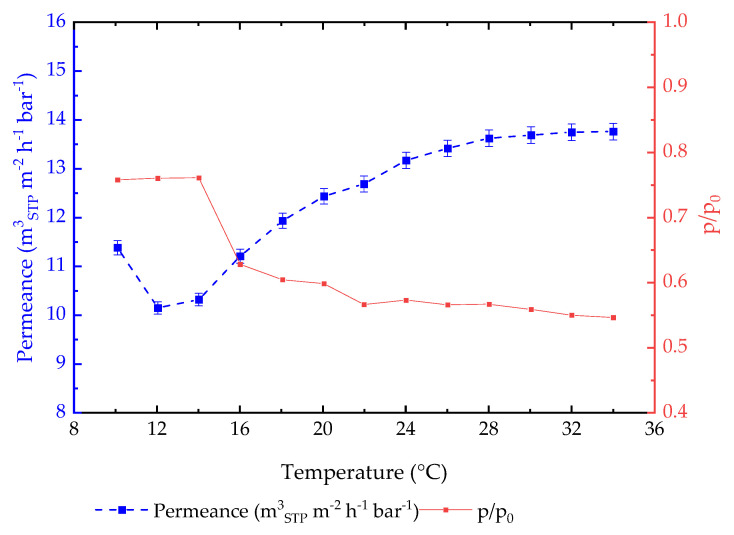
Analysis of PA77-GL TFCM peak permeance and corresponding CO_2_ feed activity in dependence on experimental feed temperature.

As mentioned above, the experiment was carried out with small steps in pressure at the same temperature and 2 °C steps in temperature between the collection of permeance data at stable feed temperature conditions. So far, only data collected at 10 °C, 20 °C, and 30 °C are presented in the Figures and analyzed. For a detailed analysis of the position of the permeance peak at different temperatures, Figure 10 demonstrates the data for all of the available temperature points for the PA77-GL TFCM membrane. For each feed temperature, the maximum values of permeance at the peak, the corresponding feed pressure, and thus, the CO_2_ feed activity were collected and presented as a function on the temperature (Figure 10). In the temperature range 10–14 °C, when poly(ethylene glycol) domains are in a semicrystalline state, the position of the permeance peak is at ca. 0.8 feed CO_2_ activity, and the permeance decreases as the temperature increases. At temperatures above 14 °C, the permeance peak was reached at a significantly lower CO_2_ feed activity, finally lowering down to a value of 0.55 at 34 °C, and the permeance gradually increased in an asymptotic manner. For the membranes under investigation, the highest achieved CO_2_ permeance was 13.8 m^3^ (STP) m^−2^ h^−1^ bar ^−1^ at the highest experimental temperature of 34 °C. The melt peak temperature for this membrane material, as reported above, is around 28 °C. Figure 10 indicates the stabilization of the maximum value for the permeance at this temperature.

For both activity and permeance curves, the change in behavior occurs in the range 14–16 °C, which is in good agreement with the observations reported earlier, where during the gas transport of the PA, TFCM was investigated at low feed pressures, and change in the Arrhenius permeance/temperature dependence associated with semicrystalline parts of the polymer was observed at temperatures of 18–20 °C [49,58]. Earlier, it was reported that the melting temperature of the poly(ethylene glycol) in the PA is 27 °C in the case of isotropic films with a ca. 100 nm thickness [27]. The difference in the melting temperatures of the bulk polymer and the polymer in the selective layer of TFCM is associated with the state of the thin layer of the polymer in the selective layer, leading to significant changes in thermal properties. Differences between the melting point observation of the current study (14–16 °C) and previous work (18–20 °C) conducted for the PA TFCM arise from the fact that, in the current study, gas transport properties were determined at much higher pressures and thus feed activities of the CO_2_, which is a plasticizing agent for the PA, can induce a decrease in the melting temperature.

#### 3.2.3. Influence of Support on TFCM Properties

The TFCMs under study have complex morphologies and consist of multiple layers. An ultrafiltration PAN layer is deposited on top of the polyester non-woven; a gutter layer of adhesive PDMS is deposited on top of the UF-PAN, followed by the selective and sometimes protective layers. The drainage of the penetrant transported through the selective layer occurs through the gutter and UF layers. It is important to investigate the possible resistance of these layers to the penetrant flow.

Figure 6 and Figure 7 show that the gas transport in UF-PAN and GL membranes is faster than in the selective membranes discussed above. The UF-PAN membrane shows a clear Knudsen-type gas flow mechanism with a CO_2_ permeance above 100 m^3^(STP) m^−2^ h^−1^ bar^−1^. 

The gas transport of CO_2_ through the GL membrane shows the same behavior as for PA77-GL, with a permeance peak at ca. 0.5 CO_2_ activity. The peak permeance of 60 m^3^(STP) m^−2^ h^−1^ bar^−1^ was observed at 20 °C for the GL membrane, followed by a sharp decrease in both the flowrate and permeance. It is interesting that in the CO_2_ activity range of 0.75–0.95, the penetrant flowrate through the membrane is independent of pressure, and above this range, it drops significantly to the point of penetrant condensation. Both pressure increase and decrease curves are shown in Figure 6. A very good agreement between these curves clearly indicates that at chosen experimental conditions (pressure step and equilibration time between experimental points), the polymer of the gutter layer reaches an equilibrium state. The exact positioning of the two curves in the activity range of 0.5–1.1 gives one proof that the observed effect of polymer/penetrant interaction in TFCMs under study is not an artefact but a real physical fact.

#### 3.2.4. Permeate Temperature Drop during Gas Transport Experiments

A gas separation membrane can be thought of as an orifice with a very small opening used for gas throttling. The gas is expanded from the high pressure side to the low-pressure side, and there is a significant drop in temperature, known as the Joule–Thomson effect [65]. The expansion of condensable gases through high-performance gas separation membranes under study should result in a significant temperature drop on the permeate side of the membrane. This drop can theoretically influence the temperature of the feed side of the selective layer.

The experimental setup enables the investigation of the temperature change in the permeate flow. The temperature sensor was immediately placed on the permeate surface of the membrane within the sintered metal support and as far away from measurement cell walls as possible to minimize the influence of the temperature outside of the cell, as shown in Figure S1 of the Supporting Information. Figure 8a shows that in the case of the PA77-GL, simultaneously with the strong variation in permeance, the temperature of the permeate stream changes. With the increase in CO_2_ permeance in the range of CO_2_ feed activity of 0–0.7, the permeate temperature drops, reaching a local minimum at the peak of permeance. In the activity range of 0.75–0.9, both the permeance and temperature change insignificantly, and in the activity range up to 1.0, both the permeance and temperature decrease. This simultaneous drop is unexpected since a reduction in the penetrant flowrate through the membrane should be accompanied by an increase in the permeate pressure in the system, where heat flow from external space is not prohibited. At an activity of one, another strong drop occurs, and afterwards, the temperature remains stable. This relationship results from the desorption process, or more precisely, the desorption enthalpy. As the amount of substance permeating through the membrane increases, the desorption of gas molecules on the permeate side of the membrane also rises. The change in temperature on the permeate side of the membrane is therefore related to the desorption rate of molecules passing through the membrane. Figure 8b shows the permeate temperature in dependence of the activity for the measurement series obtained at 10 °C, 20 °C, and 30 °C. For all measurement series, the course of the curves is similar. For higher temperatures, no such high activity was achieved in the measurement series as that at 10 °C, since the vapor pressure increases with the temperature while the maximum operating pressure is the same for all measurement series. Nevertheless, the data show that the higher the feed temperature, the more pronounced the decrease in the permeate temperature during the strong rise of the permeance. This is due to the higher permeance at higher temperatures and, therefore, the increased desorption rate.

To verify whether the observed change in the permeate temperature is a real behavior, a measurement was carried out using a PDMS thick film under the same experimental conditions as the measurements performed with TFCMs. Figure 9 shows that in this investigation, at a feed temperature of 20 °C, the permeate temperature fluctuates from the feed temperature of 0.57 °C with a linearly increasing permeate flow rate. Therefore, a technically induced temperature fluctuation can be excluded for the applied measuring range. The results show that for the measurement of the thick film membrane, no effect like the one observed for thin film membranes is observed for the measurement conditions applied.

Furthermore, Figure 8a shows the results for measurements with an increasing and decreasing feed pressure in one series of measurements. There is just a small vertical shift; it is almost negligible. The course of the curve is almost identical regardless of whether the pressure is increased or reduced during the measurement. The absence of hysteresis confirms the observation of a real relationship between the polymer and the penetrant, so measurement-based effects can be excluded. The fact that the value for permeance after pressure reduction corresponds again to the initial value at low feed pressure proves the reversible behavior and shows the stability of the membrane even at high pressures, extreme swelling, and a change in the temperature around the membrane.

Figure 8b indicates that at a feed activity of 1.0, the state of the penetrant on the feed side of the membrane changes from gaseous to liquid. The line corresponding to the feed temperature of 10 °C shows that the temperature on the permeate side of the membrane drops by 17 °C, but the gas condensation on the feed side occurs at conditions on the feed side, meaning that information on the gas state on the permeate side of the membrane is not transferred to the feed side through the selective layer. The expansion of the gas accompanied by the drop in temperature occurs on the permeate side interface of the selective layer, or, in the case of the presence of the gutter layer, on the interface between the gutter layer and porous PAN, and the penetrant flow through the selective layer is sufficiently high to keep the selective layer at feed temperature conditions. This fact can, of course, be related to the arrangement of the experimental setup used in the current study, which is similar to dead-end filtration, where there is no lateral transfer of a penetrant stream along the membrane surface on both the feed and permeate sides of the membrane. Earlier works have reported significant retentate temperature drops in comparison to the feed temperature during gas and vapor separation experiments involving full-scale membrane modules [66,67].

## 4. Conclusions

In light of the earlier reported inability of the FVM to predict gas permeances of highly efficient TFCMs at elevated partial pressures of condensable penetrants, experiments in the full range of penetrants, CO_2_, and C_2_H_6_ activities were conducted. The following TFCMs with various selective layer materials as well as support used for membrane fabrication were investigated: PA, two types of PDMS, POMS, and microporous PAN. In order to find whether permeance deviations from values predicted by FVM originate from materials or whether they are related to the TFCM morphology of thick isotropic films of PA and PDMS, they were studied under the same conditions as TFCMs.

The results obtained during sorption and gas transport experiments with isotropic films of PA and PDMS showed no deviation from behavior, which has been numerously reported in the literature.

PAN microporous support showed, at the chosen experimental conditions, a gas flow mechanism that can be characterized as a Knudsen-type flow.

The investigation of all membranes with continuous defect-free layers of rubbery polymers, namely GL, PDMS, POMS, and PA, shows the presence of a permeance peak occurring at different condensable gas activities. The highest permeance at this peak was observed for the adhesive PDMS-based GL membrane, reaching 60 m^3^(STP) m^−2^ h^−1^ bar^−1^ for CO_2_ determined at 20 °C. The feed CO_2_ activity at the peak permeance was the lowest among all the membranes under investigation.

The same peak behavior of the permeance curve in dependence of feed activity was observed for all other TFCMs, the peak position varying dependent on selective layer material, its thickness, and the temperature of the experiment.

It was found that for PA-based membranes, it is possible to observe significant changes in the permeance peak position in relation to the presence of the crystalline state of the poly(ethylene glycol) domains that are responsible for gas transport properties of the PA block copolymer.

The thermal effects on the permeate side of the membrane were investigated. It was found that an expansion of the penetrant causes a significant drop in the permeate temperature. Additionally, the temperature curve in dependence on feed gas activity is very complex and does not follow the dependence for the permeance, especially at high penetrant feed activity. A significant decrease in the permeate temperature was observed at a feed gas activity of p/po ≥ 1, i.e., the penetrant on the feed side condensed to the liquid state. The following two conclusions should be drawn from this fact: (a) multiple experiments with various membranes showed that PDMS, POMS, and PA selective layers are stable under exposure to liquid CO_2_ and C_2_H_6_; (b) sudden and significant temperature drops on the permeate side occur when the feed side pressure is equal to or exceeds the condensation pressure at the temperature of the feed side, meaning that the selective layer temperature is controlled by feed side conditions and information on temperature drops on the permeate interface of the selective layer is not transferred into the layer itself. This consideration can be valid only for the experimental conditions chosen for this study, where gas transport properties are studied as dead-end filtration and no penetrant movement occurs in the lateral direction to the membrane as in large-area membrane modules.

The reason for the permeance peak can be attributed to processes occurring within the rubbery selective layer of a TFCM during condensable penetrant transport through it: limited swelling accompanied by an increase in the free volume in accordance with the FVM; clustering of penetrant molecules, resulting in an increase in the effective kinetic diameter of a penetrant and simultaneously increased polymer swelling; free volume morphology, reaching the state when transporting through the rubbery swollen polymer matrix, according to the capillary condensation mechanism.

Further experimental and modeling efforts are necessary in order to investigate permeance peak phenomena for TFCMs with rubbery selective layers of varying thickness, glassy polymers, and polymers of high free volume. A comparison of properties should be conducted for TFCMs and integral asymmetric membranes. All this work is in progress and will be reported on shortly.

## Figures and Tables

**Table 1:** Specification of membrane samples investigated in the current study.

Sample Nomenclature	Sample Composition	Note
UF-PAN ^1^	Microporous PAN membrane on polyester non-woven.	Standard support for TFCMs, as described elsewhere [38].
PDMS1280 ^2^ -PAN	PDMS 1280 nm on UF-PAN support (Figure S2a) ^3^.	Separation layer of PDMS prepared from high-concentration solution.
GL	PDMS 600 nm on UF-PAN support.	Standard PDMS gutter layer membrane ^4^, as described elsewhere [39].
POMS6250-GL	POMS on GL membrane (Figure S2b).	POMS layer deposited using 8 wt% casting solution.
PA77-GL	PDMS as top layer on PA selective layer-coated GL membrane (Figure S2c,d).	Standard TFCM, as described elsewhere [40].
PA186-GL	PDMS as top layer on PA selective layer-coated GL membrane.	PA layer deposited using 1 wt% casting solution.
PA film	PA 388 µm, 173 µm, and 19 µm thick films.	Isotropic films with uniform thickness.
PDMS film	PDMS 285 µm and 261 µm thick films.	Isotropic films with uniform thickness.

^1^ UF-PAN support is an ultrafiltration membrane composed of a porous polyacrylonitrile layer deposited by the phase inversion method on top of a polyester non-woven. ^2^ The sample name is composed of the abbreviation for the selective layer material and the effective selective layer thickness, which is calculated as the ratio of the membrane permeance and the permeability coefficient of the selective material. ^3^ Selective layer of the PDMS1280-PAN was formed without the addition of adhesion-providing components. ^4^ The GL membrane is formed with PDMS layer containing adhesion-providing components (composition cannot be disclosed due to license infringement) with a thickness of ca. 150 nm deposited on a PAN UF support.

**Table 2:** Experimental uncertainties.

Parameter	Value, 95% Confidence
Membrane area	±0.5%
Volumetric flowrate	±0.9% of S.P. ^1^
Feed and permeate pressure determined with LEO3 sensors	0.2% F.S. ^1^
Measuring load ^1^	±0.01 mg ^1^
Pressure determined with sensor (0–50) bar	0.5% F.S. ^1^
Huber thermostat temperature stability	±0.05 °C ^1^
Temperature determined with sensor Pt100	±0.05 K at temperature below 370 K ^1^

^1^ Data provided by producer in calibration certificate.

**Table 3:** Density of isotropic films of PDMS and PA.

Material	Density in FC770 ^1^ g cm^−3^	Uncertainty g cm^−3^	Density in He ^2^ g cm^−3^	Uncertainty g cm^−3^
PDMS	1.100	±0.012	1.164	±7 × 10^−6^
PA	1.176	±0.005	1.188 ^3^	±8 × 10^−6^

^1^ Density measurements performed at room temperature; ^2^ density measurements performed at 30 °C. ^3^ As reported as well by Car et al. [27].

## Data Availability

The data presented in this study are available on request from the corresponding author.

## Supplementary Materials

The following supporting information can be downloaded at https://www.mdpi.com/article/10.3390/membranes14030066/s1. Figure S1: The scheme of the test cell build in the setup used for single gas experiments at high pressures. Figure S2: Scanning electron microscope (SEM) images of cross sections on TFCM membranes: (a) PDMS1280-PAN; (b) POMS6250-GL; (c,d) standard TFCM.

## Appendix A

The density of solid thick films was determined at room temperature as follow:

(A1) $$\rho_{S}=\frac{A}{A-B}\left(\rho_{0}-\rho_{air}\right)+\rho_{air},$$

where *ρ_S_* is the density of sample (g cm^−3^), *A* and *B* are the mass of the sample in air and in the auxiliary liquid, *ρ*_0_ is the density of the auxiliary liquid (g cm^−3^), and *ρ_air_* is the air density at room conditions (0.0012 g cm^−3^).

The density of the auxiliary liquid (FC-770) was determined using a sinker of known volume:

(A2) $$\rho_{0}=\alpha \frac{A-B}{V}+\rho_{air},$$

where *V* is the volume of the sinker (cm^3^).

The overall uncertainty of sorption measurements was estimated using the Gaussian law of propagation.

The combined standard uncertainty is the square root of the combined variance for the sorption experimental results is given by the following:

(A3) $$\sigma^{2}\left(f\left(x\right)\right)=\sum_{i=1}^{N}{\left(\frac{\partial f\left(x\right)}{\partial x_{i}}\right)}^{2}\sigma^{2}\left(x_{i}\right),$$

Here are the parameters involved in the uncertainty calculations of adsorption gas measurements: mass of polymer sample *m_S_* obtained by weighting in vacuum (g), *V_SC_* is the volume of the balance component holding the sample (cm^3^), *V_S_* is the volume of sample, and *ρ^f^* is the sorptive gas density. Each measurement point was characterized by experimental standard deviation for minimum 100 series in observation in equilibrium [68].
