# Supplementary material for: Permeance of Condensable Gases in Rubbery Polymer Membranes at High Pressure
Source: Membranes (Basel). 2024 Mar 6;14(3):66. doi: 10.3390/membranes14030066 (PMC10972516; doi:10.3390/membranes14030066)
Supplement: Supplementary file 1 [file membranes-14-00066-s001.zip › membranes-2868010-supplementary.pdf]

membranes

Supplementary Materials

Permeance of Condensable Gases in Rubbery Polymer Membranes at High Pressure

Karina Schuldt [1,2], Jelena Lillepärg [1,*], Jan Pohlmann [1], Torsten Brinkmann [1] and Sergey Shishatskiy [1]

[1] Helmholtz-Zentrum Hereon, Institute of Membrane Research, Max-Planck-Str. 1, 21502 Geesthacht, Germany
[2] Camfil GmbH, Feldstraße 26-32, 23585 Reinfeld, Germany
* Correspondence: jelena.lillepaerg@hereon.de; Tel.: +49-4152-87-2448

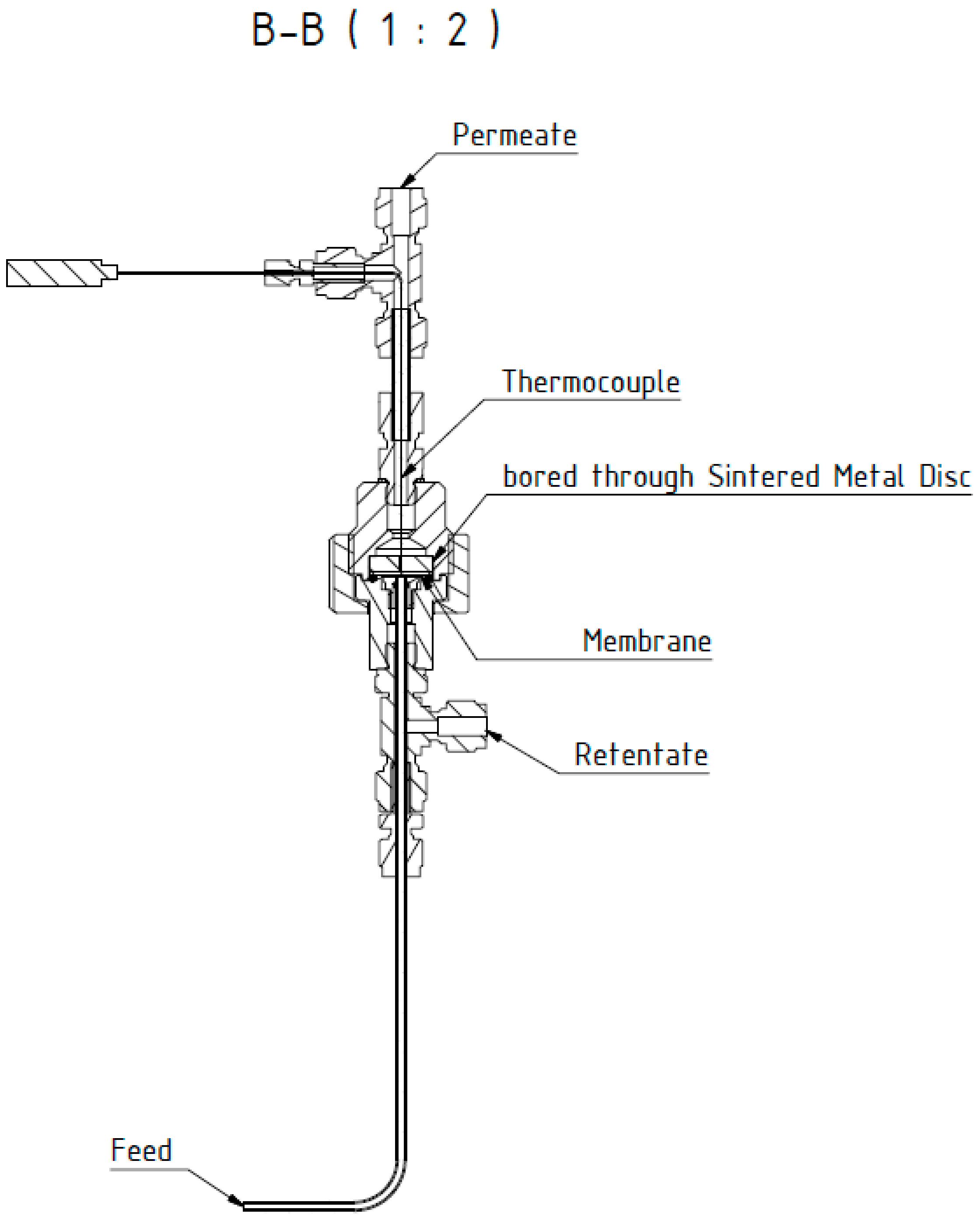

Figure S1. The scheme of the test cell build in the setup used for single gas experiments at high pressures.

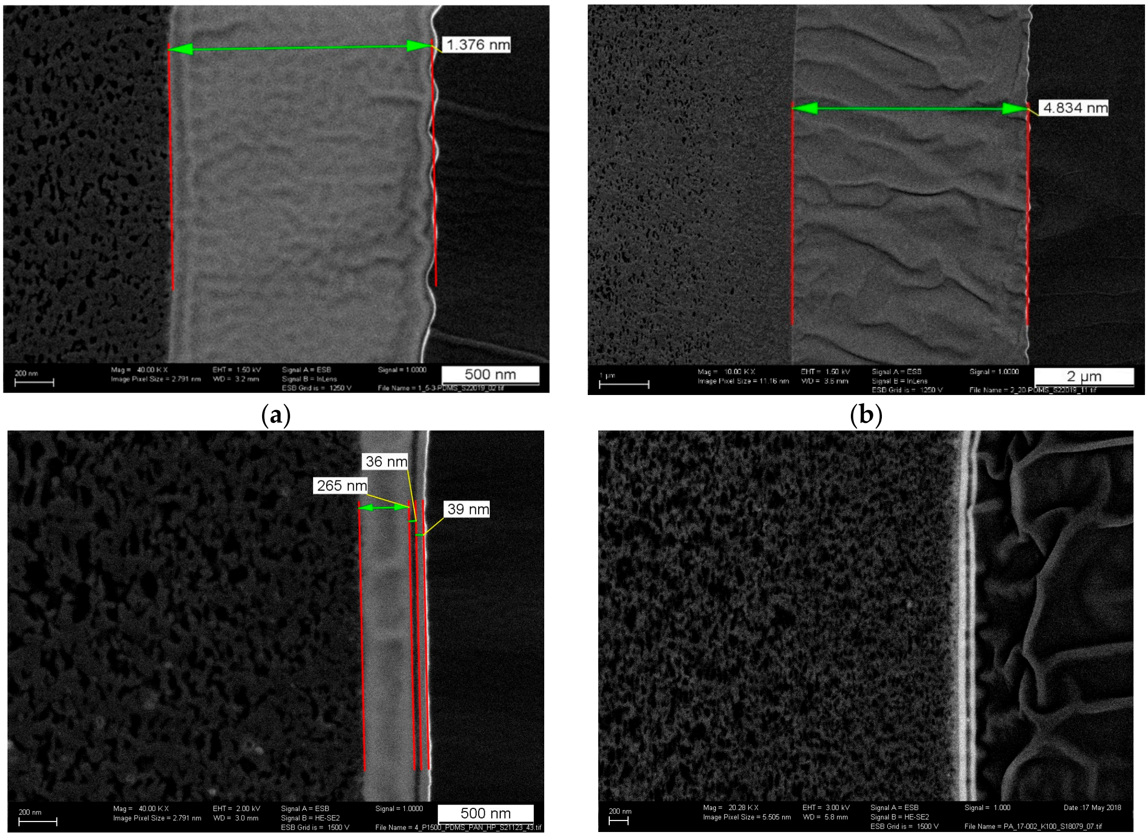

(**a**) (**b**) (**c**) (**d**)

Figure S2. Scanning electron microscope (SEM) images of cross sections on TFCM membranes: (**a**) PDMS1280-PAN; (**b**) POMS6250-GL; (**c, d**) standard TFCM.
